# Hesitancy in COVID-19 vaccine uptake and its associated factors among the general adult population: a cross-sectional study in six Southeast Asian countries

**DOI:** 10.1186/s41182-021-00393-1

**Published:** 2022-01-05

**Authors:** Roy Rillera Marzo, Waqas Sami, Md. Zakiul Alam, Swosti Acharya, Kittisak Jermsittiparsert, Karnjana Songwathana, Nhat Tan Pham, Titik Respati, Erwin Martinez Faller, Aries Moralidad Baldonado, Yadanar Aung, Sharmila Mukund Borkar, Mohammad Yasir Essar, Sunil Shrestha, Siyan Yi

**Affiliations:** 1 Department of Community Medicine, International Medical School, Management and Science University, Shah Alam, Selangor Malaysia; 2 Department of Community Medicine, Faculty of Medicine, Asia Metropolitan University, Masai, Johor Malaysia; 3 Global Public Health, Jeffrey Cheah School of Medicine and Health Sciences, Monash University Malaysia, Jalan Lagoon Selatan, 47500 Subang Jaya, Selangor, Malaysia; 4 Department of Community Medicine and Public Health, College of Medicine, Majmaah University, Almajmaah, 11952 Saudi Arabia; 5 Azra Naheed Medical College, Superior University, Lahore, 54000, Pakistan; 6 Department of Population Sciences, University of Dhaka, Dhaka, Bangladesh; 7 Department of Nursing, Nepal Health Research and Innovation Foundation, Lalitpur, Province Bagmati, Nepal; 8 College of Innovative Business and Accountancy, Dhurakij Pundit University, Bangkok, Thailand; 9 Faculty of Economics and Investment, Bangkok University, Bangkok, Thailand; 10 International University, Ho Chi Minh City, Vietnam; 11 Vietnam National University Ho Chi Minh City, Ho Chi Minh City, Vietnam; 12 Department of Public Health, Faculty of Medicine and Faculty of Graduate Studies, Universitas Islam Bandung, Bandung, Indonesia; 13 Department of Pharmacy, Faculty of Pharmacy, San Pedro College, Davao City, Philippines; 14 College of Nursing, Saint Alexius College, Koronadal, South Cotabato Philippines; 15 Medical Statistics Division, Department of Medical Research, Pyin Oo Lwin, Myanmar; 16 Institute for Population and Social Research, Mahidol University, Bangkok, Thailand; 17 Department of Economics, Sridora Caculo College, Mapusa, Goa India; 18 Kabul University of Medical Sciences, Kabul, Afghanistan; 19 Department of Pharmaceutical and Health Service Research, Nepal Health Research and Innovation Foundation, Lalitpur, Province Bagmati Nepal; 20 Saw Swee Hock School of Public Health, National University of Singapore and National University Health System, Singapore, Singapore; 21 KHANA Center for Population Health Research, Phnom Penh, Cambodia; 22 Center for Global Health Research, Touro University California, Vallejo, California, USA

**Keywords:** Immunization, Pandemic, Vaccine hesitancy, Acceptance, Multi-country study, Southeast Asia

## Abstract

**Background:**

Vaccines are effective and reliable public health interventions against viral outbreaks and pandemics. However, hesitancy regarding the Coronavirus disease (COVID-19) vaccine is evident worldwide. Therefore, understanding vaccination-related behavior is critical in expanding the vaccine coverage to flatten the infection curve. This study explores the public perception regarding COVID-19 vaccination and identifies factors associated with vaccine hesitancy among the general adult populations in six Southeast Asian countries.

**Methods:**

Using a snowball sampling approach, we conducted a descriptive cross-sectional study among 5260 participants in Indonesia, Malaysia, Myanmar, Philippines, Thailand, and Vietnam between February and May 2021. Binary logistic regression analysis with a backward conditional approach was applied to identify factors associated with COVID-19 vaccine hesitancy.

**Results:**

Of the total, 50.6% were female, and the median age was 30 years (range: 15–83 years). The majority of the participants believed that vaccination effectively prevents and controls COVID-19 (81.2%), and 84.0% would accept COVID-19 vaccines when they become available. They agreed that health providers’ advice (83.0%), vaccination convenience (75.6%), and vaccine costs (62.8%) are essential for people to decide whether to accept COVID-19 vaccines. About half (49.3%) expressed their hesitancy to receive the COVID-19 vaccines. After adjustment for other covariates, COVID-19 vaccine hesitancy was significantly associated with age, residential area, education levels, employment status, and family economic status. Participants from Indonesia, Myanmar, Thailand, and Vietnam were significantly more likely to express hesitancy in receiving COVID-19 vaccines than those from Philippines.

**Conclusions:**

In general, participants in this multi-country study showed their optimistic perception of COVID-19 vaccines’ effectiveness and willingness to receive them. However, about half of them still expressed their hesitancy in getting vaccinated. The hesitation was associated with several socioeconomic factors and varied by country. Therefore, COVID-19 vaccination programs should consider these factors essential for increasing vaccine uptake in the populations.

## Introduction

The ongoing global pandemic of coronavirus disease 2019 (COVID-19) has already infected 257 million population and of them, 5.1 million already died . Both therapeutic and non-therapeutic measures were taken to flatten the numbers of COVID-19 confirmed cases and reduce the deaths. However, the non-pharmaceutical interventions taken worldwide to tackle the pandemic have become tranquil with time [1, 2]. Therefore, it becomes essential to achieve herd immunity or implement effective vaccination. Achieving herd immunity for COVID-19 by natural means or allowing a large number of people to become infected will cause an unprecedented strain on healthcare resources and will also result in up to 30 million deaths worldwide [3]. Thus, mass vaccination has become the only way to manage COVID-19 transmission.

Vaccines other than COVID-19 are one of the most effective and reliable public health interventions ever implemented that prevent millions of deaths from viral infections every year [4–6]. Although anti-vaccination attitudes and associated misconceptions are prevalent worldwide [7, 8], vaccination programs have been developed and progressed significantly in the global health era. Currently, the vaccine from the Pfizer/BioNTech, the SII/Covishield and AstraZeneca/AZD1222 developed by AstraZeneca/Oxford, the Janssen/Ad26.COV 2.S developed by Johnson & Johnson, the Moderna COVID-19 vaccine (mRNA 1273), the Sinopharm COVID-19 vaccine from China National Biotec Group, and the Sinovac-CoronaVac are listed for WHO Emergency Use Listing (EUL). All the vaccines have some sort of mild to moderate side effects, but all of them are safe and effective (60–95%). COVID-19 vaccines save from not only infection but also severe illness and death. Though mass vaccination programs have already been started globally, the effectiveness of vaccination programs has been affected by a hesitancy to receive the vaccines in populations [9–11], where vaccine hesitancy is defined as the delay in acceptance or refusal of available vaccines [12].

The hesitancy regarding COVID-19 vaccines is prominently evident worldwide [13–15]. Studies have identified several factors associated with the COVID-19 vaccine hesitancy in different domains. The identified factors included various socioeconomic and demographic characteristics (e.g., age, sex, residence, income, occupation, and marital status) [14, 16–19] constructs of the health belief model [20, 21], constructs of theory of planned behavior and the 5c psychological antecedents [20, 22], vaccines-related knowledge [14, 23, 24], attitude towards COVID-19 vaccination [14, 18, 24], conspiracy beliefs [14, 25–27], trust and confidence [9], COVID-19 preventive behavioral practices [28–30], and the perceived safety and side effects of the vaccines [31–34]. Despite vaccine hesitancy, the demand for vaccines increases over time, and disparities in vaccine access within and across the countries are remarkable [35]. Even though the primary drivers of vaccine hesitancy are often context-specific, there are some agreements that confidence and trust in the COVID-19 vaccine play a critical role in increasing vaccine acceptance [9, 36].

COVID-19 cases have been increasing in Southeast Asian countries [37], and the COVID-19 pandemic impacted the lives of everyone, including health care workers, in many ways, including mental health [8, 38–41]. As of November 21, 2021, around 4.25, 2.82, 2.58, 2.06, 1.09, and 0.52 million confirmed cases have already been in Indonesia, Philippine, Malaysia, Thailand, Vietnam, Myanmar, respectively. The government of all the countries has been trying to mitigate the infection with several measures, including mass vaccination. Understanding vaccination-related behavior is critical in expanding the vaccine coverage to flatten the infection curve. Unfortunately, studies related to the COVID-19 vaccine hesitancy are limited in the context of these nations. As of November 21, 2021, the proportion of the general population fully vaccinated was 32.2% in Indonesia, 79.9% in Malaysia, 17.9% in Myanmar, 38.3% in Philippines, 54.73% in Thailand, and 39.6% in Vietnam [37]. Though started with AstraZeneca in the first phase, Pfizer, Sinovac, and Covovax vaccines are available in east Asian countries. The hesitancy to receive the COVID-19 vaccine may pose critical challenges in the fight against the pandemic and the global shortage of vaccines. To address this gap, we conducted a multi-country study to assess the perception of the COVID-19 vaccine effectiveness, acceptance, and hesitancy in the context of Southeast Asian countries. We also explored factors associated with the hesitation in the vaccine uptake.

## Methods

### Study design and sites

This descriptive cross-sectional study was conducted in six Southeast Asian countries i.e., Indonesia, Malaysia, Myanmar, Philippines, Thailand, and Vietnam for 4 months from February to May 2021.

### Participants and sampling

The target participants were adult citizens from the participating countries aged 18 years and above, who could read and understand local languages or English. Due to the limitations in employing face-to-face methods during the outbreak, the survey was prepared in a Google form and disseminated to the participants using a snowball sampling method. First, we recruited 50 primary participants and asked them to share the questionnaire link to individuals in their social networks who met the inclusion criteria. We chose these social media platforms, because they are widely used across socio-demographic characteristics. The response rate ranged from 30–45%.

### Data collection procedures

We distributed the questionnaire using personal contacts using word of mouth or emails and through web-based applications and social media, such as Facebook, Instagram, LinkedIn, Telegram, Twitter, and WhatsApp. Participants were reminded to respond only once. We employed unique identifiers for use only in a single account by settings that allow only one response per user. In addition, the Google form will not allow another entry from the same Google Account. Participants were ensured the confidentiality and privacy of their responses to reduce potential bias introduced by self-reported data.

### Tool development and measures

We developed the questionnaire through participatory discussion with the research team of participating countries. Through Zoom meetings, the principal author discussed research objectives and methodology with all country representatives. The questionnaire was initially developed in English and translated into local languages. Then, the questionnaire was back-translated, pre-tested, and revised by the research team in the individual country. A group of expert panels in the respective countries which included psychiatrists, clinical psychologists, physicians, clinicians and public health experts translated and culturally validated into their national. Pilot testing comprised of 15 participants in each country to test face validity and 50 participants in each country to test the internal consistency. The Cronbach's alpha value ranging from 0.824 to 0.925 indicated that the questionnaire has a good to excellent internal consistency across all countries. It took approximately 10 mins to complete the survey.

The questionnaire had 15 items divided into two sections, namely, Section A had nine items and Section B had six questions regarding factors influencing the acceptability of COVID-19 vaccination. The socio-demographic characteristics of the participants were age (continuous), sex (male, female), place of residence (rural, urban), the education level (illiterate, secondary, post-secondary education, tertiary education), employment status (employed, student, unemployed), marital status (never married, married, widowed/divorced/separated), and family economic status (low, medium, high). The economic status was classified according to income classification from Department of Statistics for each country.

We used yes/no questions to assess the participants’ perceived COVID-19 vaccine effectiveness, acceptance, and factors believed to be essential for deciding whether to accept COVID-19 vaccines. Participants were asked whether they think COVID-19 vaccination can effectively prevent and control COVID-19. They were also asked whether they would accept COVID-19 vaccines when they become available. We asked whether the participant agreed that vaccination convenience (methods, frequency, distance to vaccination sites), health providers’ advice, and costs of vaccines are essential for deciding whether to accept COVID-19 vaccines. Hesitancy in COVID-19 vaccine uptake was measured by asking whether the participant would take COVID-19 vaccines as soon as they become available in the country.

### Statistical analyses

We used Statistical Product and Service Solutions (SPSS) 26.0 (IBM Corp., Armonk, N.Y., USA) for data analyses. One-sample Kolmogorov–Smirnov test was used to assess the normality of age distribution, and it was non-normally distributed. Therefore, median and range were used as a measure of central tendency. Categorical variables are presented as frequencies and percentages. Pearson’s Chi-square test was used to observe the association between socio-demographics and the COVID-19 vaccine-related variables and between-country differences. The Bonferroni-adjusted post hoc test was applied for significant results in the Pearson’s Chi-square tests. After fulfilling the assumptions (relationship between variables and absence of multicollinearity), we conducted binary logistic regression analyses with a backward conditional approach to explore the relationship between vaccine hesitancy (yes, no) and socio-demographic characteristics controlling for the covariates included in the models. All variables associated with vaccine hesitancy in bivariate analyses at a level of *p* value < 0.05 were included in the multivariable regression analyses.

### Ethics

Each study country representative obtained ethical clearance. The format of informed consent forms for all selected studies adhered to the guidelines recommended by the SOMREC which, at the minimum, stipulate inclusion of sections on purpose of the research, study procedures, discomforts and risks, potential benefits, privacy and confidentiality, compensation for participation, voluntary participation, investigators’ contact information for questions about study, and ethics committee contact for questions about rights and welfare of participants.

## Results

Table 1 shows the socio-demographic characteristics of the participants. A total of 5260 participants completed the questionnaire—339 from Indonesia, 1273 from Malaysia, 300 from Myanmar, 311 from Philippines, 2367 from Thailand, and 670 from Vietnam. The median age of the participants was 30 years (range: 15–83 years). Slightly more than half of the participants were female (50.6%) and never married (55.4%). About two-thirds of the participants were employed (61.7%), and 69.6% resided in urban areas. More than half of the participants (55.6%) had tertiary education. Almost half of the participants (46.6%) reported medium family economic status, and 45% were from Thailand.

**Table 1:** Socio-demographic characteristics of participants (*n* = 5260)

Socio-demographic characteristics	Number (%)
Female	2660 (50.6)
Urban residence	3661 (69.6)
Employment status	
Employed	3243 (61.7)
Student	1826 (34.7)
Unemployed	191 (3.6)
Education level	
≤ Primary	38 (0.7)
Secondary	814 (15.5)
Post-secondary	1484 (28.2)
Tertiary	2924 (55.6)
Family economic status	
Low	866 (16.5)
Medium	1944 (37.0)
High	2450 (46.6)
Marital status	
Never married	2912 (55.4)
Married	2189 (41.6)
Widowed/divorced/separated	159 (3.0)
Country of residence	
Indonesia	339 (6.4)
Malaysia	1273 (24.2)
Myanmar	300 (5.7)
Philippines	311 (5.9)
Thailand	2367 (45.0)
Vietnam	670 (12.7)

As shown in Table 2, 81.2% of the participants agreed that vaccination could effectively prevent and control COVID-19, and 84.0% would accept the vaccines when they become available. The majority believed that vaccination convenience (75.6%), health providers’ advice (83.0%), and costs of vaccines (62.8%) are essential for deciding whether to accept COVID-19 vaccines. However, about half (50.7%) still expressed their hesitation to take the COVID-19 vaccines.

**Table 2:** Overall perceived effectiveness, acceptance, and determinants of COVID-19 vaccine uptake decision-making (*n* = 5260)

	Number (%)
Agreed that vaccines are can effectively prevent and control COVID-19	4271 (81.2)
Would accept COVID-19 vaccines when available	4418 (84.0)
Believed that vaccination convenience is important for deciding whether to accept vaccines	3976 (75.6)
Believed that health providers’ advice is important for deciding whether to accept COVID-19 vaccines	4367 (83.0)
Believed that costs of the vaccines are important for deciding whether to accept COVID-19 vaccines	3303 (62.8)
No hesitancy in receiving COVID-19 vaccines	2592 (49.3)

Table 3 shows that males were significantly more likely to agree that vaccines could effectively prevent and control COVID-19, responded that they would accept the vaccines when they become available, and believed that health providers’ advice and costs of vaccines are important for deciding whether to accept COVID-19 vaccines than females. Participants living in urban areas were significantly more likely to agree that vaccines can effectively prevent and control COVID-19, responded that they would accept the vaccines when they become available, and believed that vaccination convenience, health providers’ advice, and costs of vaccines are important for deciding whether to accept COVID-19 vaccines than those living in rural areas. Participants living in rural areas were significantly more likely to express hesitancy in receiving COVID-19 vaccines than those living in urban areas.

**Table 3:** Perceived effectiveness, acceptance, and determinants of COVID-19 vaccine uptake decision-making by socio-demographic characteristics (*n* = 5260)

Socio-demographic characteristics	Agreed that vaccines are effective to prevent and control COVID-19	Would accept COVID-19 vaccines when available	Believed that convenience is important for people to decide whether to accept vaccines	Believed that health providers’ advice is important for people to decide whether to accept vaccines	Believed that cost of the vaccines is important for people to decide whether to accept vaccines	Hesitant to receive COVID-19 vaccines
Sex						
Female	2088 (78.5)	2178 (81.9)	1991 (74.8)	2175 (81.8)	1615 (60.7)	1342 (50.4)
Male	2183 (84.0)^a^	2240 (86.2)^a^	1985 (76.3)	2192 (84.3)^a^	1690 (65.0)^a^	1250 (48.1)
*p* value	< 0.001	< 0.001	0.21	0.01	0.001	0.09
Residential area						
Rural	1187 (74.2)	1217 (76.1)	1016 (63.5)	1139 (71.2)	766 (47.9)	901 (56.3)^a^
Urban	3084 (84.2)^a^	3201 (87.4)^a^	2960 (80.9)^a^	3228 (88.2)^a^	2539 (69.4)^a^	1691 (46.2)
*p* value	< 0.001	< 0.001	< 0.001	< 0.001	< 0.001	< 0.001
Education level						
≤ Primary	36 (94.7)	35 (92.1)	17 (44.7)	20 (52.6)	5 (13.2)	34 (89.5)^a^
Secondary	1151 (77.6)	1142 (77.0)	1050 (70.8)	1090 (73.5)	840 (56.6)	799 (53.9)^a^
Post-secondary	540 (66.3)	531 (65.2)	520 (63.9)	534 (65.6)	422 (51.8)	422 (51.8)^a^
Tertiary	2544 (87.0)^a^	2710 (92.7)^a^	2389 (81.7)^a^	2722 (93.1)^a^	2038 (69.7)^a^	1337 (45.7)
*p* value	< 0.001	< 0.001	< 0.001	< 0.001	< 0.001	< 0.001
Employment status						
Employed	2429 (74.9)	2596 (80.0)	2222 (68.5)	2515 (77.6)	1800 (55.5)	1603 (49.4)
Student	1679 (91.9)^a^	1653 (90.5)^a^	1598 (87.5)^a^	1692 (92.7)^a^	1386 (75.9)^a^	871 (47.7)
Unemployed	163 (85.3)	169 (88.5)	156 (81.7)	160 (83.8)	119 (62.3)	118 (61.8)^a^
*p* value	< 0.001	< 0.001	< 0.001	< 0.001	< 0.001	< 0.001
Marital status						
Widowed/divorced/separated	111 (69.8)	120 (75.5)	95 (59.7)	115 (72.3)	96 (60.4)	88 (55.4)
Married	1692 (77.3)	1813 (82.8)	1540 (70.4)	1753 (80.1)	1225 (56.0)	1069 (48.8)
Single	2468 (84.8)^a^	2485 (85.3)^a^	2341 (80.4)^a^	2499 (85.8)^a^	1984 (68.1)^a^	1435 (49.3)
*p* value	< 0.001	< 0.001	< 0.001	< 0.001	< 0.001	0.29
Family economic status						
High	751 (86.7)^a^	807 (93.2)^a^	600 (69.3)	733 (84.6)^a^	423 (48.8)	459 (53.1)^a^
Low	1507 (77.5)	1535 (79.0)	1474 (75.8)	1574 (81.0)	1283 (66.0)^a^	1013 (52.1)^a^
Medium	2013 (82.2)	2076 (84.7)	1902 (77.6)^a^	2060 (84.1)	1599 (65.3)	1120 (45.7)
*p* value	< 0.001	< 0.001	< 0.001	0.009	< 0.001	< 0.001
Country of residence						
Indonesia	306 (7.2)	264 (6.0)	339 (8.5)	315 (7.2)	181 (5.5)	264 (10.2)
Malaysia	1221 (28.6)	1223 (27.7)	1218 (30.6)	1241 (28.4)	1162 (35.2)	487 (18.8)
Myanmar	255 (6.0)	271 (6.1)	260 (6.5)	259 (5.9)	211 (6.4)	187 (7.2)
Philippines	277 (6.5)	254 (5.7)	289 (7.3)	292 (6.7)	254 (7.7)	106 (4.1)
Thailand	1624 (38.0)	1806 (40.9)	1550 (39.0)	1797 (41.1)	1385 (41.9)	976 (37.7)
Vietnam	588 (13.8)	600 (13.6)	320 (8.0)	463 (10.6)	112 (3.4)	572 (22.1)
*p* value	< 0.001	< 0.001	< 0.001	< 0.001	< 0.001	< 0.001

^a^Bonferroni adjusted post-hoc comparisons (exact *p* values are mentioned in the text)

Participants with tertiary education were significantly more likely to agree that vaccines can effectively prevent and control COVID-19, responded that they would accept the vaccines when they become available, and believed that vaccination convenience, health providers’ advice, and costs of vaccines are important for deciding whether to accept COVID-19 vaccines than participants with lower education. Participants with lower education were significantly more likely to express hesitancy in receiving COVID-19 vaccines than participants with tertiary education. Compared to unemployed and employed participants, students were significantly more likely to agree that vaccines can effectively prevent and control COVID-19, responded that they would accept the vaccines when they become available, and believed that vaccination convenience, health providers’ advice, and costs of vaccines are important for deciding whether to accept COVID-19 vaccines. Unemployed participants were significantly more likely to express hesitancy in receiving COVID-19 vaccines than students and employed participants.

Never-married participants were significantly more likely to agree that vaccines can effectively prevent and control COVID-19, responded that they would accept the vaccines when they become available, and believed that vaccination convenience, health providers’ advice, and costs of vaccines are important for deciding whether to accept COVID-19 vaccines than married and widowed, divorced or separated participants. Participants with a high family economic status were significantly more likely to agree that vaccines can effectively prevent and control COVID-19, responded that they would accept the vaccines when they become available, and believed that health providers’ advice is important for deciding whether to accept COVID-19 vaccines than participants with a low and medium family economic status. Participants with a medium family economic status were significantly more likely to believe that vaccination convenience is important for deciding whether to accept COVID-19 vaccines than participants with a low and high family economic status. Participants with a low family economic status were significantly more likely to believe that vaccine costs are important for deciding whether to accept COVID-19 vaccines than participants with a medium and high family economic status. Participants with a low and high family economic status were significantly more likely to express hesitancy in receiving COVID-19 vaccines than participants with a medium family economic status. The differences between countries were all statistically significant.

Table 4 presents the association between countries and COVID-19 vaccine effectiveness, acceptance, convenience, recommendation, price, and hesitancy. Results showed a significant association between all vaccine factors and countries (*p* < 0.001), respectively.

**Table 4:** Association between COVID-19 (effectiveness, acceptance, convenience, recommendation, price, and hesitancy) with Countries

COVID-19	Countries						
	Indonesia *N* = 339 *n* (%)	Malaysia *N* = 1273 *n* (%)	Myanmar *N* = 300 *n* (%)	Philippines *N* = 311 *n* (%)	Thailand *N* = 2367 *n* (%)	Vietnam *N* = 670 *n* (%)	*p* value
Effectiveness							< 0.001^a^
No	33 (3.3)	52 (5.3)	45 (4.6)	34 (3.4)	743 (75.1)	82 (8.3)	
Yes	306 (7.2)	1221 (28.6)	255 (6.0)	277 (6.5)	1624 (38.0)	588 (13.8)	
Acceptance							< 0.001^a^
No	75 (8.9)	50 (5.9)	29 (3.4)	57 (6.8)	561 (66.6)	70 (8.3)	
Yes	264 (6.0)	1223 (27.7)	271 (6.1)	254 (5.7)	1806 (40.9)	600 (13.6)	
Convenience							< 0.001^a^
No	0 (0.0)	55 (4.3)	40 (3.1)	22 (1.7)	817 (63.6)	350 (27.3)	
Yes	339 (8.5)	1218 (30.6)	260 (6.5)	289 (7.3)	1550 (39.0)	320 (8.0)	
Recommendation							< 0.001^a^
No	24 (2.7)	32 (3.6)	41 (4.6)	19 (2.1)	570 (63.8)	207 (23.2)	
Yes	315 (7.2)	1241 (28.4)	259 (5.9)	292 (6.7)	1797 (41.1)	463 (10.6)	
Price							< 0.001^a^
No	158 (8.1)	111 (5.7)	89 (4.6)	57 (2.9)	982 (50.2)	588 (28.5)	
Yes	181 (5.5)	1162 (35.2)	211 (6.4)	254 (7.7)	1385 (41.9)	112 (3.4)	
Hesitancy							< 0.001^a^
No	75 (2.8)	786 (29.5)	113 (4.2)	205 (7.7)	1391 (52.1)	98 (3.7)	
Yes	264 (10.2)	487 (18.8)	187 (7.2)	106 (4.1)	976 (37.7)	572 (22.1)	

^a^Significant at 5% level of significance

Table 5 shows factors associated with hesitancy in COVID-19 vaccine uptake in the logistic regression model. After adjustment, having no hesitation was significantly associated with living in rural areas (AOR: 1.40, 95% CI: 1.24–1.59), lower education (AOR: 7.74, 95% CI: 2.72–22.05 for illiterate, AOR: 1.19, 95% CI: 1.01–1.41 for secondary education, and AOR: 1.29, 95% CI: 1.13–1.47 for post-secondary relative to tertiary education), family economic status (AOR: 1.23, 95% CI 1.09–1.39 for lower and AOR: 1.39, 95% CI: 1.19–1.63 for higher relative to medium-income), and employment status (AOR: 1.21, 95% CI 1.03–1.42 for being employed and AOR: 1.85, 95% CI: 1.14–2.60 for being unemployed relative to being students). Compared to those from Philippines, participants from Indonesia (AOR: 6.81, 95% CI: 4.81 9.64), Myanmar (AOR 3.20, 95% CI: 2.30–4.46), Thailand (AOR: 1.06, 95% CI: 1.06–1.74), and Vietnam (AOR: 11.28, 95% CI: 8.22–1.50) were significantly more likely to express no hesitancy in receiving COVID-19 vaccines.

**Table 5:** Factors associated with hesitancy in COVID-19 vaccine uptake in logistic regression model (*n* = 5260)

Variables in the model	AOR (95% CI)	*P* value
Age	0.99 (0.98–0.99)	< 0.001
Residential area		
Urban	Reference	
Rural	1.40 (1.24–1.59)	
Education level		
Tertiary	Reference	
≤ Primary	7.74 (2.72–22.05)	< 0.001
Secondary	1.19 (1.01–1.41)	0.04
Post-secondary	1.29 (1.13–1.47)	< 0.001
Family economic status		
Medium	Reference	
Low	1.23 (1.09–1.39)	0.001
High	1.39 (1.19–1.63)	< 0.001
Employment status		
Student	Reference	
Employed	1.21 (1.03–1.42)	0.02
Unemployed	1.85 (1.14–2.60)	1.85
Country of residence		
Philippines	Reference	
Indonesia	6.81 (4.81–9.64)	< 0.001
Malaysia	1.20 (0.92–1.56)	0.17
Myanmar	3.20 (2.30–4.46)	< 0.001
Thailand	1.36 (1.06–1.74)	0.02
Vietnam	11.28 (8.22–1.50)	< 0.001

AOR, adjusted odd ratio; CI, confidence interval

## Discussion

Our multi-country study of six countries of the Southeast Asian region provides essential insight into the perception of COVID-19 vaccines, acceptability, hesitancy, and factors associated with hesitation in the vaccine uptake. Most participants believed that vaccination effectively prevents and controls COVID-19 and would accept COVID-19 vaccines when they become available. They agreed that health providers’ advice, vaccination convenience, and vaccine costs are essential for deciding whether to accept COVID-19 vaccines. However, about half expressed their hesitancy to receive the COVID-19 vaccines. The highest rate of vaccine hesitancy has been observed in Russia (72%), whereas the lowest in Vietnam (27%) [9].

We have identified several socio-demographic factors associated with hesitancy in COVID-19 vaccine uptake, including age, residential area, education level, family economic status, employment status, and country of residence. The existing studies from Southeast Asian countries [19, 42] also show that the older populations are more likely to express their hesitation in receiving the vaccines than the younger populations. In addition, participants from low and high family economic backgrounds were more likely to show uncertainty in receiving COVID-19 vaccines than those with medium family financial status. Previous studies have reported several factors that may explain the populations’ hesitancy in COVID-19 vaccine acceptance. The factors include lower economic level [43], concerns about the possibly damaging outcome of the COVID-19 vaccines to developing babies in the womb [44], conspiracy beliefs regarding the COVID-19 vaccine might cause infertility and miscarriages [45], and less perceived susceptibility [46].

Place of residence was one of the significant factors that may determine COVID-19 acceptance and uptake. In this study, urban residents were more likely to support COVID-19 vaccines’ effectiveness and uptake. They were more likely to believe that vaccination convenience, advice from health providers, and vaccine costs are important for people deciding whether to receive the vaccines than rural residents. Similarly, rural residents had a higher level of hesitation in the COVID-19 vaccine than urban residents. These findings are similar to other studies conducted in Bangladesh and Philippines [14]. Higher levels of accessibility, affordability, education, and standard of living are related to vaccine acceptability among people living in urban areas. Having more exposure to the different sources of information, urban residents can create more comprehensive access to more accurate information through media and other reliable sources regarding vaccines. Exposure to negative information about the vaccines was associated with a high level of vaccine hesitancy in Philippines [47]. There is the need for accurate information on the COVID-19 vaccine, which is very important for its proper management [48].

Education level was also associated with hesitancy in COVID-19 uptake in this study. People with tertiary education were more likely to support COVID-19 vaccines’ effectiveness and uptake than those with lower education. They were also more likely to believe that vaccination convenience, health providers’ advice, and costs of vaccines are important for people to decide whether to receive COVID-19 vaccines. Similarly, people with lower education more hesitated when asked whether they would accept COVID-19 vaccines than people with tertiary education. Higher educated populations generally possess better knowledge about the vaccines and vaccination process [49], which creates more heightened awareness regarding the risks and benefits of the vaccination. The level of hesitancy decreases when the level of knowledge about the COVID-19 vaccine and its associated processes increases [14]. Better knowledge of the vaccination process was a significant factor associated with vaccine hesitancy in previous studies in Bangladesh, Malaysia, India, Kenya, Myanmar, and Thailand [14, 16, 50].

## Strengths and limitations

To our knowledge, this is the first multi-country study examining the factors associated with COVID-19 vaccine hesitancy in Southeast Asia. We collected data from a large sample in six countries assessing vaccine effectiveness, acceptance, and hesitation in various populations from different contexts, cultures, and backgrounds. Despite these strengths, this study has several limitations. Response biases could be one of the critical limitations of the study. In addition, data were collected using the snowball technique, which could hamper the heterogeneity in the sample. Another significant limitation is the representativeness of the sample population. A higher proportion of the sampled population were highly educated and residing in urban areas. Since, hesitancy was slightly lower among educated and urban residents, overrepresentation of these groups could lead to underestimation of vaccine hesitancy.

## Conclusions

This study provides a crucial understanding of the populations’ perception required to design effective COVID-19 vaccine programs in Southeast Asia. Participants in this multi-country study generally showed their optimistic perception of COVID-19 vaccines’ effectiveness and willingness to receive them. However, about half of them still expressed their hesitancy in getting vaccinated. The hesitation was associated with several socioeconomic factors and varied by country. COVID-19 vaccination promotion campaign should consider these factors as essential elements for increasing vaccine uptake in the populations in the region. Further studies on COVID-19 vaccine acceptance and hesitancy should be a priority. We can use the studies' findings to inform contextualized vaccination programs and information-sharing, ultimately resulting in increased confidence in and uptake of the available vaccines.

## Data Availability

The data relating to this manuscript are available upon request.
